# Favourable mid‐ to long‐term clinical and functional outcomes and low redislocation rates following derotational distal femoral osteotomy for the treatment of recurrent patellofemoral instability

**DOI:** 10.1002/jeo2.70629

**Published:** 2026-01-13

**Authors:** Peter Rab, Andrea Achtnich, Maximilian Hinz, Florian B. Imhoff, Elmar Herbst, Luca Grüning, Moritz Brunner, Sebastian Siebenlist, Romed P. Vieider

**Affiliations:** ^1^ Department of Sports Orthopaedics, TUM University Hospital Technical University of Munich Munich Germany; ^2^ Department of Orthopaedics and Traumatology University Hospital Basel Basel Switzerland; ^3^ Department of Trauma, Hand and Reconstructive Surgery University of Muenster Muenster Germany; ^4^ Department of Trauma Surgery Regensburg University Medical Center Regensburg Germany

**Keywords:** derotational distal femoral osteotomy, femoral antetorsion, patellar dislocation, patellofemoral instability, torsional deformity

## Abstract

**Purpose:**

To assess the mid‐ to long‐term clinical and functional outcomes and return to sports following derotational distal femoral osteotomy (DDFO) for the treatment of recurrent patellofemoral instability and associated increased femoral antetorsion.

**Methods:**

Patients who underwent DDFO as well as concomitant procedures between 2007 and 2016 were eligible for inclusion. Recurrent instability, complications, revision surgery and return to sports rates were evaluated after a minimum follow‐up of 5 years. Kaplan–Meier survival analysis was performed for the risk of recurrent instability or undergoing further surgery other than hardware removal. Patient‐reported outcome measures (PROMs; Visual Analog Scale [VAS] for pain, Tegner Activity Scale [TAS], Kujala score, Banff Patellofemoral Instability Instrument [BPII] 2.0 and Patellofemoral Instability‐Return to Sport after Injury [PFI‐RSI] scale) were recorded.

**Results:**

Of 44 knees in 42 patients eligible for inclusion, 25 knees in 24 patients (64% female) with a mean age of 27.3 ± 9.0 years at the time of surgery were available for follow‐up at a mean of 8.7 ± 2.7 years (follow‐up: 57%). Two patients (5%) underwent early revision surgery due to loss of osteosynthesis at the distal femur. Three patients (12%) reported subjective recurrent instability at a median of 3.9 (3.5–7.0) years post‐operatively. The Kaplan–Meier estimator showed a survival probability free of recurrent instability and revision (except hardware removal) of 87% (95%‐confidence interval 76%–100%) at 5 years. At final follow‐up, patients reported good knee function (Kujala score: 78.5 ± 16.6), knee‐related quality of life (BPII: 68.3 ± 22.3), psychological readiness to return to sport (PFI‐RSI: 73.3 [43.8–90.0]), and moderate activity levels (TAS: 4.0 [3.0–4.0]). The majority of patients returned to sports (84%).

**Conclusion:**

DDFO provided effective treatment for recurrent patellofemoral instability associated with increased femoral antetorsion. Favourable clinical and functional outcomes as well as high return to sport rates were observed at a mid‐ to long‐term follow‐up.

**Level of Evidence:**

Level IV, retrospective case series.

**Abbreviations**
95%‐CI: 95% confidence interval
BMI: body mass index
BPII: Patellofemoral Instability Instrument [BPII] 2.0
DFO: distal femoral osteotomy
femAT: femoral antetorsion
IQR: interquartile range
mLDFA: mechanical lateral distal femoral angle
mMPTA: medial proximal tibial angle
MPFL: medial patellofemoral ligament
PFI‐RSI: Patellofemoral Instability‐Return to Sport after Injury Scale
SD: standard deviation
TAS: Tegner Activity Scale
TTO: tibial tubercle osteotomy
TT–TG: tibial tubercle–trochlear groove
VAS: Visual Analog Scale

## INTRODUCTION

Patellofemoral instability (PFI) is a common condition with a multifactorial aetiology, predominantly affecting young females [56]. The consequences of untreated PFI include persistent pain, instability, reduced quality of life and subsequent post‐traumatic osteoarthritis [1, 19, 42]. Coronal limb malalignment, torsional deformities of the femur and tibia, tibiofemoral torsion, trochlear dysplasia, patella alta, or disrupted and weakened medial retinaculum complex, including the medial patellofemoral ligament (MPFL) and the vastus medialis obliquus, may contribute to PFI [9, 28, 41, 47]. While favourable outcomes have been reported for isolated MPFL reconstruction and is considered standard of care [8, 29], PFI requires a comprehensive risk factor analysis and individualised treatment [9, 48]. In this context, increased femoral antetorsion (femAT) has been demonstrated to be a risk factor for the development of PFI [21, 32], as well as a risk factor for recurrent instability, if not surgically addressed [24, 34, 54].

For patients with PFI and increased femAT, derotational distal femoral osteotomy (DDFO) has been demonstrated as a suitable treatment option, yielding favourable short‐ to mid‐term outcomes and low rates of recurrent instability [17, 25, 44, 49, 50]. Moreover, superior patient‐reported outcomes in patients with increased femAT who underwent patella stabilising procedures with concomitant DDFO have been reported compared to patients without concomitant correction of increased femAT [51, 54]. However, there is a paucity of data regarding mid‐to long‐term clinical and functional outcomes as well as return to sports (RTS) and work following DDFO for the treatment of PFI associated with increased femAT.

Therefore, the primary purpose of this study was to evaluate the clinical and functional outcomes, as well as recurrent instability rates, in patients who underwent individualised surgical treatment, including DDFO for the treatment of PFI, at mid‐ to long‐term follow‐up. It was hypothesised that DDFO for the treatment of PFI would yield low recurrent instability rates and satisfactory patient‐reported outcomes which could be maintained in the long term. The secondary aim of this study was to assess RTS rates and return to work rates following DDFO, with the hypothesis that high rates of RTS and return to work would be achieved at follow‐up.

## MATERIALS AND METHODS

This retrospective case series was conducted according to the Declaration of Helsinki and approved by the institutional review board of the Technical University of Munich (reference: 110/18 S). Consecutive patients who underwent DDFO for the treatment of recurrent PFI (≥ 2 patellar dislocations) and increased femAT (> 20°), as well as concomitant procedures between 2007 and 2016 who participated in a previous study investigating short‐term outcomes of DDFO were eligible for inclusion [22]. Concomitant procedures included additional correction of valgus malalignment, reconstruction of the MPFL, tibial tubercle osteotomy (TTO), trochleoplasty, and treatment of (osteo‐)chondral lesions. Patients with less than 5 years of follow‐up and those who were not available for the previously published short‐term follow‐up [22] were excluded. In line with the previous study assessing short‐term outcomes following DDFO, patients younger than 16 years at the time of surgery, those with open growth plates, posttraumatic deformities, prior osteotomy, or isolated frontal plane correction at the distal femur were also excluded [22]. Patients were contacted via phone or mail exclusively for this study. All patients provided informed consent. Chart review was performed to record patient demographics such as age at surgery, gender, body mass index (BMI), smoking status, and surgical characteristics.

### Pre‐operative assessment and surgical planning

To evaluate the individual risk factors contributing to PFI, all patients underwent a standardised pre‐operative clinical and radiological assessment as published previously [22]. The clinical evaluation included the assessment of patellar tracking, apprehension, range of motion of the hip and knee joint as well as gait analysis. Coronal alignment of the lower limb was evaluated using weight‐bearing whole‐leg anteroposterior radiographs, which were analysed for parameters such as the femorotibial angle, mechanical lateral distal femoral angle, and mechanical medial proximal tibial angle [43]. In cases with coronal malalignment, digital pre‐operative planning was carried out using a digital planning software (mediCAD® version 5.1, Hectec, Altdorf, Germany). Patellar height was measured using the Caton‐Deschamps Index (CDI) on lateral knee radiographs [7]. Trochlear dysplasia was classified based on the Dejour classification [31]. Non‐contrast magnetic resonance imaging of the lower extremity was performed to assess patellar tilt, femAT, TT–TG distance, and trochlear dysplasia. FemAT was measured according to the method described by Schneider et al. [37].

DDFO was generally indicated in case of increased femAT, with the threshold for surgical indication ranging between > 20° and > 30°, influenced by surgeon‐specific preferences involved in clinical practice [17, 22]. Concomitant procedures were performed according to the patient‐individual risk‐factors recorded preoperatively. Concomitant varization at the distal femur was performed in case of a femoral valgus deformity with a femorotibial angle ≥ 3°. A TTO was performed in patients with TT–TG > 20 mm or CDI ≥ 1.2. In patients exhibiting a Dejour type B or D trochlear dysplasia, a positive J‐sign and apprehension ≥ 60° knee flexion, a trochleoplasty was performed. Lastly, concomitant MPFL reconstruction was performed in patients with residual intraoperative lateral lateralisation tendency of the patella after other surgical corrections.

### Surgical technique

In all patients, diagnostic arthroscopy was performed to assess patellar tracking, cartilage lesions, and the medial retinacular complex. DDFO was performed using as biplanar supracondylar technique via a standardised lateral subvastus approach with the aim of physiological femAT, as previously described [16, 17]. In cases with concomitant correction of valgus malalignment, the additional correction was performed as previously described by adding a lateral opening wedge with the aim of neutral alignment [16, 17]. An internal plate fixation system with locking screws (Tomofix distal femoral plate, DePuy Synthes, Umkirch, Germany; or PEEK distal femoral plate, Arthrex Inc., FL, USA) was used to secure the DDFO. Trochleoplasty was performed according to Bereiter [3, 4], and TTO was performed as described by Fulkerson [13]. Following DDFO, patellar tracking was assessed, and MPFL reconstruction was performed using the ipsilateral gracilis tendon as described by Schöttle et al. [38] in case of high lateralisation tendency.

### Post‐operative rehabilitation

Postoperatively, weight bearing was limited to 20 kg for 6 weeks and was gradually increased thereafter. In patients who underwent isolated DDFO, ROM was not limited. In patients who underwent concomitant MPFL reconstruction, flexion was limited to 90° for 6 weeks using a knee brace (M.4 s®, medi Bayreuth, Germany). In case of concomitant trochleoplasty, flexion was limited to 60° and extension to 20° for 2 weeks, which was gradually increased thereafter. For patients who underwent concomitant TTO, flexion was limited to 20° for 2 weeks, which was gradually increased after. Physical therapy was started on the first post‐operative day and comprised passive mobilisation and gait training, with active exercises added according to the concomitant procedures performed. Return to cycling, running, sports‐specific training and competition were decided based on patient‐specific findings during clinical assessment starting from three months post‐operatively and gradually increased in intensity and complexity.

### Outcome assessment

Clinical and functional outcomes were evaluated preoperatively and after a minimum follow‐up of 5 years. Recurrent instability (including subjective subluxations as well as clinically and radiographically confirmed redislocations), complications, revision surgery and RTS and return to work rates were recorded. Patient‐reported outcome measures (PROMs; Visual Analog Scale [VAS] for pain, Tegner Activity Scale [TAS] [45], Kujala score [26], Banff Patellofemoral Instability Instrument [BPII] 2.0 [27] and Patellofemoral Instability‐Return to Sport after Injury [PFI‐RSI] scale [18, 20]) were recorded. Preoperatively, VAS for pain and TAS were obtained. At final follow‐up, VAS for pain, TAS, BPII, Kujala score, and PFI‐RSI were evaluated. Moreover, a 10‐point numeric rating scale was used to assess patient satisfaction (10, very satisfied; 1, very unsatisfied) [2]. Additionally, RTS was defined as the return to the preinjury sports discipline at any level, and the return to the pre‐injury level of sports was defined as the return to the preinjury level of sport. Moreover, the reasons for a reduced level of sports were obtained. Furthermore, the return to work and return to previous level of work rates were assessed. Finally, recurrent instability during follow‐up and complications that required revision surgery were recorded.

### Statistical analysis

Statistical analysis was performed using SPSS 26.0 (IBM‐SPSS, New York, USA), RStudio (RStudio Public Benefit Corporation, Boston, USA) and R version 4.4.0 (R Foundation for Statistical Computing, Vienna, Austria). Normality of the distribution was assessed using histograms and the Shapiro–Wilk test. Normally distributed data were reported using mean ± standard deviation (SD), whereas median (interquartile range) was used to report non‐normally distributed data. Categorical data were presented using absolute values and percentages. To compare normally distributed data across time points, the paired t‐test was applied, and the Wilcoxon signed rank test was employed for non‐normally distributed data. Kaplan–Meier survival analysis was performed for the risk of recurrent instability or undergoing further surgery other than hardware removal, displayed with 95% confidence interval (95% CI). Power analysis was not conducted because this study continues a prior study and the procedure is relatively rare with limited cases [22]. A significance level of < 0.05 was set, and all *p*‐values were two‐tailed.

## RESULTS

**Figure 1:**
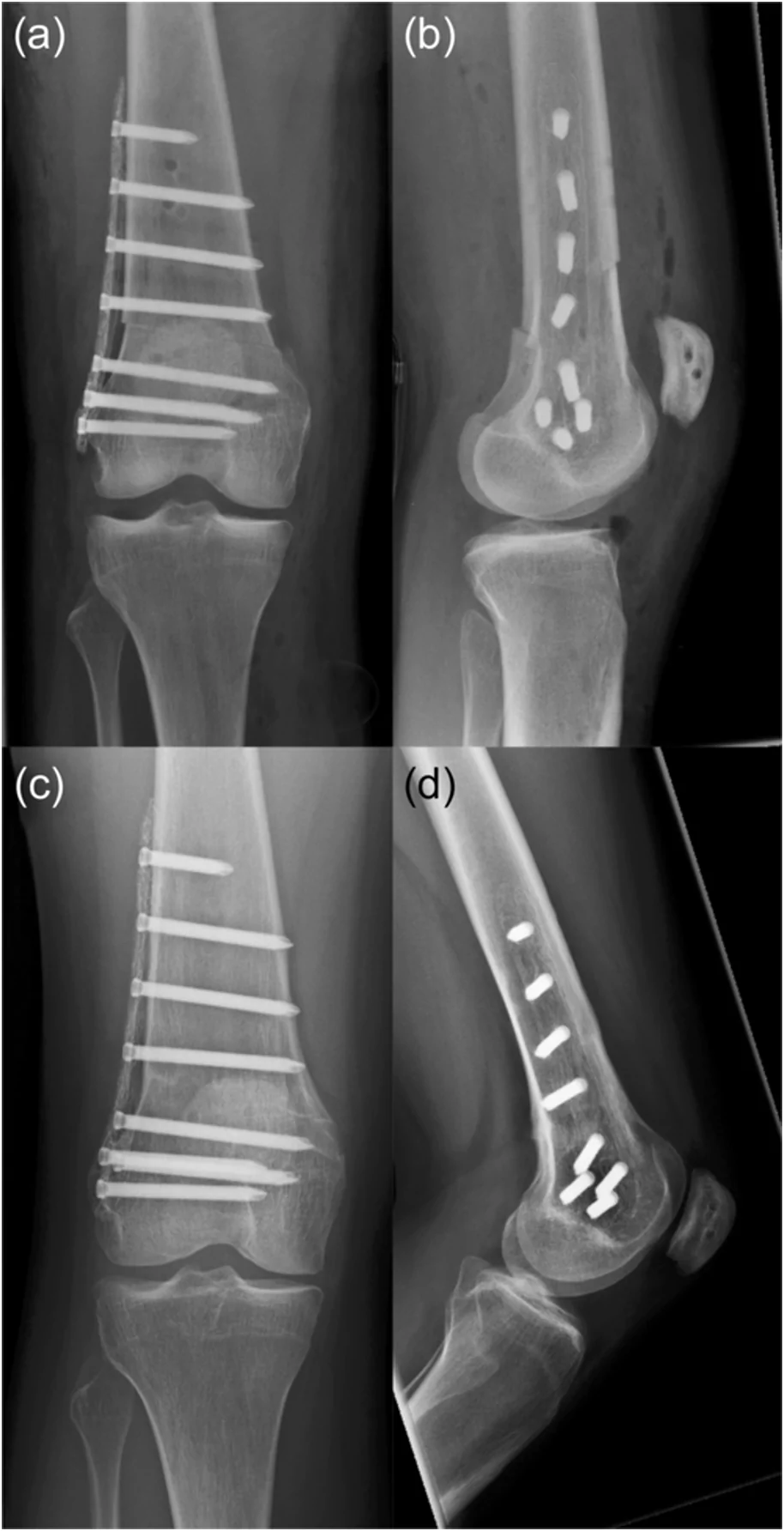
Clinical case of a 21‐year‐old female patient who underwent derotational distal femoral osteotomy with concomitant medial patellofemoral ligament reconstruction for the treatment of recurrent patellofemoral instability. (a, b) Plain radiographs 2 days post‐operatively; (c, d) plain radiographs 1‐year post‐operatively showing consolidated osteotomy.

**Figure 2:**
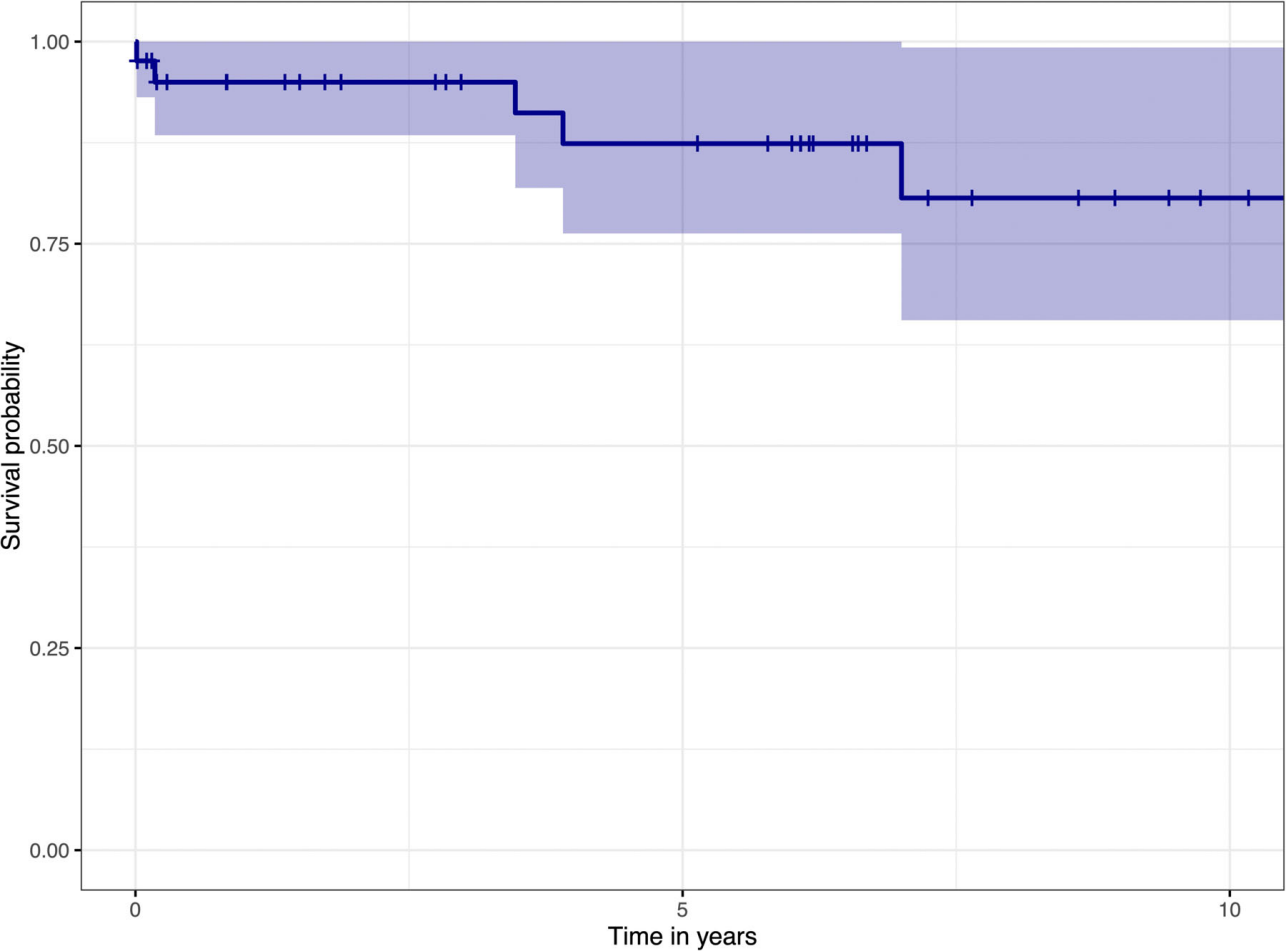
Kaplan–Meier survival analysis showing survival probability free from recurrent instability and revision, except for hardware removal.

Of 44 knees in 42 patients enrolled in the previous study, 25 knees in 24 patients (64% female) with a mean age of 27.3 ± 9.0 years at the time of surgery were available for follow‐up at a mean of 8.7 ± 2.7 years (follow‐up: 57%). Of these, concomitant procedures were performed in 21 patients (84%), of whom 6 (24%) underwent these procedures as part of a planned, staged approach (Table 1, Figure 1). Two patients (5%) underwent early revision surgery due to loss of osteosynthesis at the distal femur at 0.6 and 9.2 weeks post‐operatively. Three patients (12%) reported subjective recurrent instability at a median of 3.9 (3.5–7.0) years post‐operatively. All three patients had undergone concomitant MPFL reconstruction. Recurrent instability could not be verified clinically or radiographically, as these patients did not present for assessment at the time of recurrence. None of these patients underwent revision surgery. The Kaplan–Meier estimator showed a survival probability free of recurrent instability and revision (except hardware removal) of 87.4% (95% CI 76.3%–100%) at 5 years and 80.7% (95% CI 65.5%–99.3%) at 8 years (Figure 2). During the follow‐up period, 19 patients (76%) underwent plate removal 3.4 ± 1.2 years after the index procedure.

**Table 1:** Patient demographics procedures performed and pre‐operative radiographic parameters of patients available at final follow‐up.

Patient demographics (*n* = 25)	
Age at surgery, years	27.3 ± 9.0
Sex, female:male (% female)	16:9 (64.0)
BMI (kg/m^2^)	26.0 ± 5.3
Active smoker/former smoker, *n* (%)	4/6 (16.0/24.0)
Procedures performed, *n* (%)	
DDFO	13 (52.0)
Isolated	1 (4.0)
With concomitant procedures	12 (48.0)
MPFL reconstruction	8 (8.7)
Trochleoplasty	1 (4.0)
TTO	1 (4.0)
TTO and MPFL reconstruction	2 (8.0)
Derotational and varus‐producing DFO	12 (48.0)
Isolated	3 (12.0)
With concomitant procedures	9 (36.0)
MPFL reconstruction	5 (25.0)
TTO	1 (4.0)
Trochleoplasty and MPFL reconstruction	2 (8.0)
Cartilage therapy	1 (4.0)
Radiographic parameters	
FemAT, mean degrees ± SD	31.3 ± 8.8
Femorotibial angle, median degrees (IQR)	1.9 (0.3–3.5)
mLDFA, median degrees (IQR)	86.3 (84.4–87.4)
mMPTA, median degrees (IQR)	88.1 (86.4–90.0)

Abbreviations: BMI, body mass index; DDFO, derotational distal femoral osteotomy; IQR, interquartile range; MPFL, medial patellofemoral ligament; mLDFA, mechanical lateral distal femur angle; mMPTA, mechanical medial proximal tibia angle; SD, standard deviation; TTO, tibial tubercle osteotomy.

**Figure 3:**
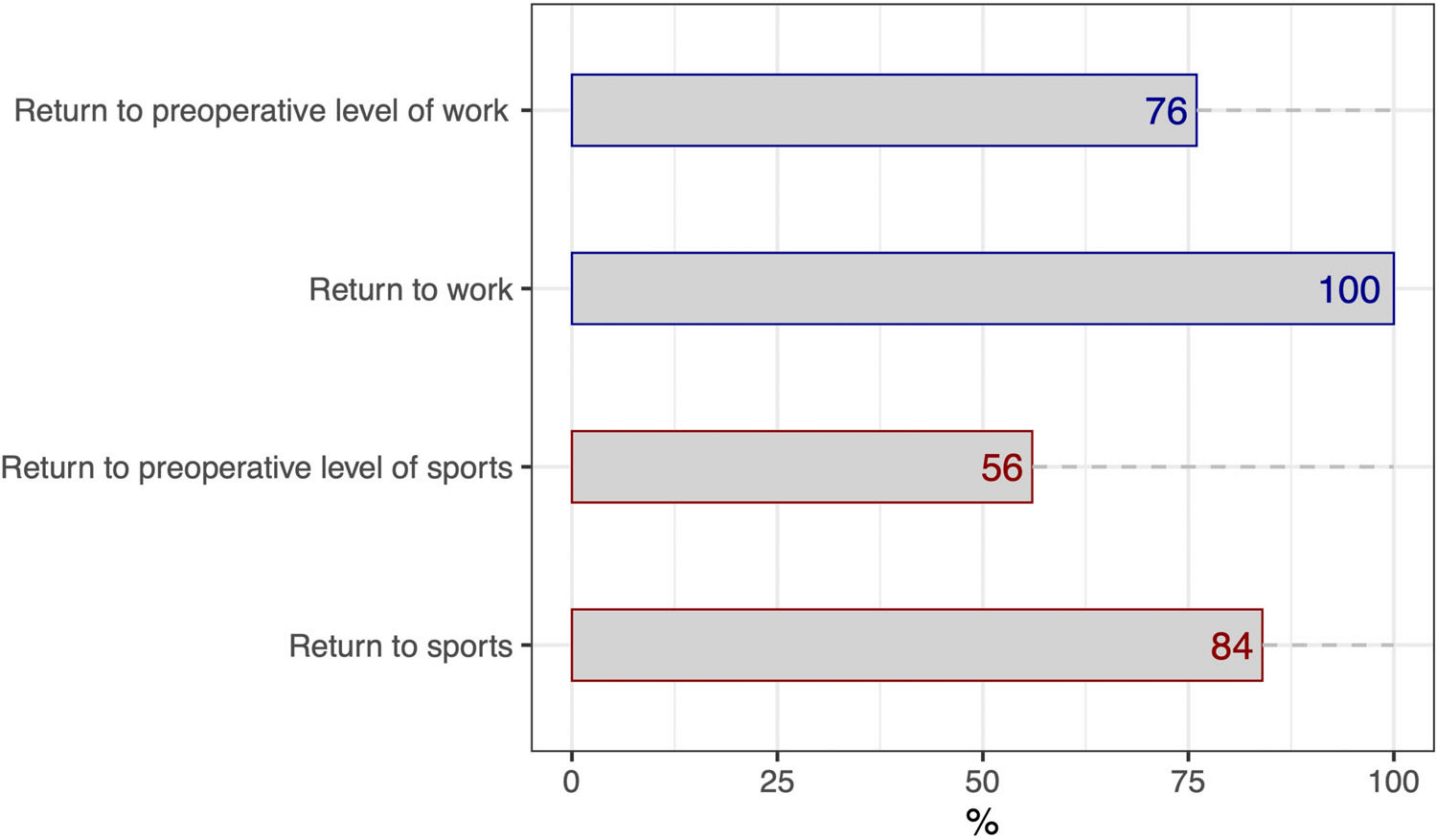
Return to sports and work rates following derotational distal femoral osteotomy.

At final follow‐up, favourable knee function, knee‐related quality of life, patient satisfaction and psychological readiness to return to sports were reported (Table 2). No significant changes were detected for VAS (*p* = 0.95) and TAS (*p* = 0.77). In total, 21 patients (84%) reported to have achieved RTS, of which 14 patients (56%) had returned to their pre‐operative level of sports. The reported reasons for a reduced sporting ability were fear of reinjury (*n* = 8; 73%), missing confidence in the knee joint (*n* = 8; 73%), knee pain (*n* = 7; 64%), subjective instability (*n* = 7; 64%), and other reasons not related to the knee joint (*n* = 7; 27%). All patients returned to work, of whom the majority of patients (*n* = 19; 76%) reported a return to their previous level of work (Figure 3).

**Table 2:** Clinical and function outcome parameters preoperatively of patients available at final follow‐up.

Measurement parameters (*n* = 25)	Pre‐operative	At final follow‐up	*p* value
VAS for pain at rest, median (IQR)		1.0 (0–2.0)	
VAS for pain during activity, median (IQR)	3.0 (0.5–6.0)	2.0 (1.0–5.0)	0.95
TAS, median (IQR)	4.0 (3.0–4.5)	4.0 (3.0–4.0)	0.77
Kujala score, mean ± SD		78.5 ± 16.6	
BPII, mean ± SD		68.3 ± 22.3	
PFI‐RSI [IQR]		73.3 (43.8–90.0)	
Patient satisfaction, median (IQR)		7 (3–9)	
Return to sports, *n* (%)		21 (84.0)	
Return to pre‐operative level of sports, *n* (%)		14 (56.0)	
Return to work, *n* (%)		25 (100)	
Return to previous level of work, *n* (%)		19 (76.0)	

Abbreviations: BPII, Patellofemoral Instability Instrument 2.0; IQR, interquartile range; PFI‐RSI, Patellofemoral Instability‐Return to Sport after Injury scale; SD, standard deviation; TAS, Tegner Activity Scale; VAS, Visual Analog Scale.

## DISCUSSION

The most important findings of the present study were favourable knee function as well as low pain levels in patients who underwent DDFO as well as concomitant intra‐ and extra‐articular procedures for the treatment of PFI at mid‐to long term follow‐up. A high rate of return to sports (84%) was observed, but only about half of the patients achieved a return to their pre‐operative level of sports (56%). Moreover, all patients returned to work, with the majority of patients (76.0%) reporting a return to their previous level of work.

The favourable clinical and functional outcomes, as well as the RTS and return to work rates, are consistent with the findings reported in previous studies evaluation outcomes following DDFO, indicating positive short‐to‐mid‐term patient‐reported outcomes, excellent healing rates, and improvements in gait and activity levels [17, 22, 25, 35, 46, 50, 55]. To the knowledge of the authors, this study represents the longest follow‐up in patients of patients undergoing DDFO, with studies reporting long‐term outcomes remaining scarce [25, 46, 50]. While an increased femAT > 20° is a well‐established risk factor for PFI [23], and isolated MPFL reconstruction has been demonstrated to be inadequate for restoring normal patellar tracking in biomechanical studies [24, 40], clinical findings have been less consistent. In contrast, several studies have observed inferior clinical and functional outcomes in cases of elevated femAT treated with isolated MPFL reconstruction compared to MPFL reconstruction with concomitant DDFO [12, 15, 50, 51, 53]. Conversely, one study suggested that isolated MPFL reconstruction may be sufficient for treating patients with PFI, reporting no significant differences in clinical or functional outcomes between those with and without increased femAT [5]. However, the follow‐up period was limited to a minimum of 24 months, and the study was likely underpowered to detect differences in recurrent instability rates [5]. Substantial heterogeneity exists regarding the threshold of femAT, ranging from 20° to 30°, as well as in measurement methods and concomitant procedures performed, which may explain the differences in reported clinical outcomes [25, 46, 50].

The current study observed a recurrent instability rate of 12%, which is higher than the generally reported cumulative recurrent instability rate of 1.1%, ranging from 0% to 13.3% [25, 46, 50]. While substantial heterogeneity in femAT cut‐off, surgical technique, and moderate sample sizes may limit the aggregation of existing studies, the subjective redislocations in this study were observed at 3.9 (3.5–7.0) years post‐operatively, indicating that recurrent instability may still occur later in follow‐up. Moreover, the reported recurrent instability events could not be verified clinically or radiographically and may, in part, represent episodes of subluxation rather than true redislocation. This may be confounded by an increase in residual laxity following MPFL reconstruction over time [39, 46, 50, 52]. Therefore, comparative studies with adequate power, standardised measurement protocols, and long follow‐up are necessary to assess recurrent instability rates and long‐term outcomes following DDFO. Furthermore, a high rate of subsequent plate removal (76%) was observed. While this aligns with the current literature on derotational or varus osteotomy at the distal femur [10, 14], future research is warranted to determine which implant types can be kept without causing irritation or pain.

In the current study, while 84% of patients achieved RTS, only 56% returned to their pre‐operative level of sports, with fear of reinjury, missing confidence in the knee, knee pain, and subjective instability as the most common reasons for reduced sportive ability. While evidence specifically addressing RTS following DDFO is limited, these findings align with previously reported RTS rates for complex PFI procedures, despite the limitations of substantial heterogeneity and small sample sizes [6, 11, 30, 33, 36]. Moreover, the observed rates of return to the pre‐operative level of sports are lower, ranging from 45% to 84% [30, 33, 36]. In accordance with the findings of the present study, previous research has reported fear of reinjury as the most common reason for a reduced post‐operative sporting ability, followed by lack of confidence, persistent instability, and pain [18, 33, 36]. These findings are underlined by the previously established importance of psychological factors regarding RTS after surgery for PFI [18, 20, 33, 36]. While further research is needed to further establish RTS and psychological readiness following DDFO, surgeons should consider assessing psychological readiness in post‐operative care and provide thorough counselling to patients regarding realistic expectations for RTS.

The findings of this study should be interpreted considering several limitations. First, the moderate sample size and follow‐up rate (57%) may have led to a selection bias and led to underestimation of recurrent instability rates. As the indications for this procedure are rare, its results provide valuable information about long‐term outcomes. Second, this study included only PROMs without objective clinical or radiological assessments, and the absence of certain pre‐operative PROMs limits pre‐ to post‐operative comparability and reporting of patients achieving the minimal clinically important difference. Third, the present cohort is heterogenous in terms of age and concomitant procedures. This, however, represents clinical practice when treating patients with PFI in combination with high‐grade femoral antetorsion. Finally, the paucity of post‐operative radiographic and torsional analysis did not permit assessment of achieved torsional correction and further explorative analysis.

## CONCLUSION

DDFO provided effective treatment for recurrent patellofemoral instability associated with increased femoral antetorsion. Favourable clinical and functional outcomes as well as high return to sport and work rates were observed at a mid‐to long‐term follow‐up.

## AUTHOR CONTRIBUTIONS

All authors contributed to the study conception and design. Data collection was performed by Moritz Brunner and Luca Grüning, and statistical analysis and data interpretation was performed by Peter Rab, Maximilian Hinz, and Romed P. Vieider. The first draft of the manuscript was written by Peter Rab. All authors commented on previous versions of the manuscript. All authors read and approved the final manuscript.

## CONFLICT OF INTEREST STATEMENT

Peter Rab has received funding from DePuy Mitek Sports Medicine Inc. for a fellowship. Andrea Achtnich has received consultant fee payments from Arthrex GmbH and is a member of the AO sports steering board and board of the Gesellschaft für Arthroskopie und Gelenkchirurgie (AGA). Florian Imhoff is a consultant for Arthrex GmbH and Enovis. Elmar Herbst is Deputy Editor‐in‐Chief for the Knee Surgery, Sports Traumatology and Arthroscopy (KSSTA). Sebastian Siebenlist has received consultant fee payments from Arthrex GmbH, KLS Martin Group and medi GmbH & Co. KG unrelated to this study. The other authors declare no conflict of interest.

## ETHICS STATEMENT

The present study was approved by the institutional review board of the Technical University of Munich (reference: 110/18 S) and conducted according to the Declaration of Helsinki. Informed consent was obtained from all patients.

## Data Availability

All data generated or analysed during this study are included in this published article.
